# Antibodies to Heat Shock Proteins 90α and 90β in Psoriasis

**DOI:** 10.1007/s00005-020-00573-7

**Published:** 2020-04-01

**Authors:** Aleksandra Damasiewicz-Bodzek, Magdalena Szumska, Krystyna Tyrpień-Golder

**Affiliations:** Department of Chemistry, Faculty of Medical Sciences in Zabrze, Medical University of Silesia, Katowice, Poland

**Keywords:** Psoriasis, Hsp90α, Hsp90β, Anti-Hsp90α antibodies, Anti-Hsp90β antibodies

## Abstract

One of many hypotheses of psoriasis pathogenesis supposes an overexpression of heat shock proteins (Hsps) in different skin layers and systemic immunologic response to them. Hsp90 is one of the most abundant chaperone in eukaryotic cells. The number of studies concerning the role of Hsp90 and anti-Hsp90 antibodies in etiopathogenesis of various diseases is also constantly expanding. Still, there are not many reports concerning potential involvement of this Hsp family or anti-Hsp90 immunization in pathomechanism of psoriasis. The aim of the study was the estimation of anti-Hsp90α and anti-Hsp90β IgG antibodies in the sera of the psoriatic patients at different phases of disease activity in comparison to the sera of healthy individuals. The study material consisted of sera from psoriasis patients (*n* = 80) in active phase and in the remission phase and healthy individuals (*n* = 80). Concentrations of anti-Hsp90α and anti-Hsp90β IgG antibodies were determined using ELISA technique. In the patients with psoriasis (both in the active phase of the disease and in the remission phase) concentrations of anti-Hsp90α antibodies were significantly higher than in healthy individuals and they correlated positively with psoriasis area severity index values. The mean concentrations of anti-Hsp90β antibodies in the psoriatic patients and healthy controls were comparable. The obtained results indicate an existence of increased immunological response to Hsp90α in psoriasis. It may suggest the role of the extracellular form of this chaperone and/or anti-Hsp90α antibodies in etiopathogenesis of this dermatosis. The inhibition of Hsp90α may represent a novel therapeutic approach to treat psoriasis.

## Introduction

The etiopathogenesis of psoriasis is very complex and has not been fully known, which results in serious therapeutic problems. One of many hypotheses of psoriasis pathogenesis supposes an overexpression of heat shock proteins (Hsps) in different skin layers and systemic immunologic response to these proteins (Ghoreishi 2000). Heat shock proteins are cellular chaperones (prevents irreversible denaturation and aggregation of cellular proteins destroyed by stress conditions). They are also responsible for the balance of intracellular redox potential, stabilization of the cytoskeleton and regulation of complicated processes of cellular proliferation and differentiation, apoptosis, and oncogenesis. Heat shock proteins are highly conserved protein found in very different species such as bacteria and mammals. During evolution both their amino acid sequences and their functions did not change (Ang et al. 1991; Ciocca and Calderwood 2005; Jolly and Morimoto 2000; Kregel 2002; Lanneau et al. 2008; Smith et al. 1998). As immunodominant molecules, Hsps can also stimulate the immune system and induce B- and T-cell reactivity. Antibodies against these proteins can be found in healthy people and they can be treated as a part of natural autoantibodies spectrum. However, their overexpression can reflect increased expression of shock proteins caused by specific pathological conditions under which cells are found (exposure to heat, oxygen deficiency, xenobiotics, oxidative stress, inflammation, infection, etc.) (Rowley and Karopoulos 1996; Wu and Tanguay 2006). Immunological response in people can take the form of autoagression (response to homological Hsps) or the form of reactivity to heterological Hsps, for example bacteria-derived. Since the Hsps are highly conserved, anti-Hsp antibodies or T cells arising in response to foreign antigens may also cross-react with autoantigens. For that reason the possible role of the shock proteins in etiopathogenesis of various diseases is suspected. It is well-known that the Hsps or immunological response against Hsps takes part in development of many diseases, among others autoimmune diseases, cancers, neurodegenerative diseases, atherosclerosis, etc. (Luo et al. 2010; Ranford and Henderson 2002; Wu and Tanguay 2006; Young et al. 1993). It is also believed that immune reaction to the Hsp epitopes may be a link between infections and autoimmune diseases (Zugel and Kaufmann 1999), thus it could be implicated in the pathogenesis of such diseases as psoriasis, where response to the Hsps may be related to microbial foci complicating the course of this dermatosis (Besgen et al. 2010; Cancino-Diaz et al. 2004; Ishikara et al. 2000; Rambukkana et al. 1993).

Individual Hsp families differ in molecular mass, cellular localization, and specific functions among each other (Kaźmierczuk and Kiliańska 2009; Kregel 2002). The Hsp90 is one of the most abundant chaperone proteins in eukaryotic cells, accounting for 2–3% of cellular proteins under physiological conditions, and up to 6% of cellular proteins in tumor cells (Sahu et al. 2012). In human cells, Hsp90 occurs in two cytoplasmic isoforms: Hsp90α and Hsp90β, which are 86% identical and 93% similar in their amino acid sequences (Csermely et al. 1998; Sreedhar et al. 2004; Wright et al. 2004). Molecular chaperone Hsp90, like other Hsps, plays an essential role in the stress tolerance. It protects nascent cellular proteins from aggregation, participates in their maturation, enables stabilization, and transport to place of destination. On the other hand, Hsp90 performs some more specific functions, also under normal conditions. It is the main element of fundamental cellular processes, such as hormonal signaling, cell cycle control or activation of many regulatory proteins. Hsp90 modulates the stability and/or transport of diverse critical cellular proteins (more than hundred transcription factors, growth factors, and protein kinases) (Jolly and Morimoto 2000; Picard 2002; Pratt and Toft 2003; Sreedhar et al. 2004), takes part in processes of DNA and RNA metabolism (Dezwaan and Freeman 2008; Powers and Workman 2007) and is also engaged in oncogenesis (in facilitating malignant transformation and maintaining malignant phenotype) (Bagatell and Whitesell 2004; Staufer and Stoeltying 2010).

The list of Hsp90-dependent signaling proteins (“clients”) is expanding rapidly. Disruption of this chaperone function by mutations or treatment with inhibitors leads to multiple physiological defects in cells, which consequently can lead to defects at tissue or organism level (Picard 2002; Pratt and Toft 2003; Young et al. 2001). The number of studies concerning the role of Hsp90 and anti-Hsp90 immune response in etiopathogenesis of many various diseases is also constantly expanding. Still, there are not many reports about the potential involvement this Hsp family or anti-Hsp90 immunization in the pathomechanism of psoriatic lesions formation. Therefore, the aim of the study was the estimation of anti-Hsp90α and anti-Hsp90β antibodies levels in the sera of the psoriatic patients at different phases of disease activity in comparison to their concentrations in the sera of healthy individuals.

## Materials and Methods

The study included archival sera samples stored deep frozen. The studied group consisted of 80 patients with psoriasis (35 women and 45 men, mean age 37.1 ± 10.8 years). These patients had suffered from psoriasis for 122 ± 97 months (range 1–372 months). In two cases it was the first disease manifestation. The blood samples were taken twice: in active phase of disease [mean psoriasis area severity index (PASI): 25.7 ± 15.1] before any anti-psoriasis treatment and in the remission phase (remission obtained by different therapeutic methods). PASI below 3 or its reduction by over 90% was the remission criterion. The mean time to remission was 42 ± 26 days. Patients with any concurrent diseases were excluded from the study. The control group consisted of 80 healthy volunteers (40 women and 40 men), at a comparable age (36.1 ± 10.1 years; *p* > 0.05), in whom no familial predisposition to psoriasis was found. Blood samples were taken at fasting, from elbow veins. The sera obtained by centrifugation was stored at −85 °C until tests were performed. The study protocol was accepted by the Local Bioethical Commission of Silesian Medical University in Katowice (Poland). All participants were informed and signed content to participate in the study.

Anti-Hsp90α and anti-Hsp90β IgG concentrations in the tested sera were determined using ELISA technique. Human recombinant Hsp90α and Hsp90β (Enzo Life Sciences, USA) were used as antigens. ELISA plates (Maxisorp, Nunc, Denmark) were coated over 24 h at 4 °C with the antigen solution at 2 mg/mL in 50 mmol/L carbonate buffer (pH 9.6). Then the plates were rinsed with PBST (phosphate buffered saline containing 0.5 ml/L Tween 20; pH 7.2) and next the stabilizing solution (BioStab Immunoassay Satabilizer, Fluka) was added. After next 2 h, 1% bovine albumin solution (BSA) in PBS was added. A day later solution was removed and plates were stored at 4 °C. The tested sera were diluted 400× in 0.5% BSA in PBST and incubated on the plates at 4 °C for 24 h. After rinsing (four-times in PBST) bound antibodies were detected using goat anti-human IgG conjugated with horseradish peroxidase (Sigma, USA). Incubation with 6000 times diluted conjugate was performed at 25 °C for 2 h. Then the plates were rinsed again and the reaction was developed using substrate–tetramethylbenzidine (Sigma, USA). After next 20–30 min reaction was stopped by adding 0.5 mol/L sulphuric acid. Absorbance was measured at 450 nm (reference wave: 630 nm) using PowerWave XS Reader (BioTek, USA), and results were calculated using KCJunior software (BioTek, USA). Calibrations were performed using pooled sera originating from approximately 100 healthy blood donors. The 400-times dilution was accepted as 100 arbitral units/mL (AU/mL) (calibrating curve consisted of seven standards: 0–400 AU/mL). Coefficients of intra-assay variation for both ELISA methods were below 10%.

The obtained results were presented using basic parameters of descriptive statistics, such as mean value and standard deviation. Normal distribution of data was measured using Shapiro–Wilk test. Independent data between the group of psoriatic patients (in both stages of the disease) and the control group were compared using non-parametric Kolmogorov–Smirnov and *U* Mann–Whitney tests. Wilcoxon matched-pairs test was used to compare dependent data between the period of active disease and remission period. The Spearman’s rank test was used for correlations. The *p* < 0.05 was considered as statistically significant. Calculations were performed with STATISTICA for Windows 12.0 software (StatSoft, Cracow, Poland).

## Results

**Fig. 1:**
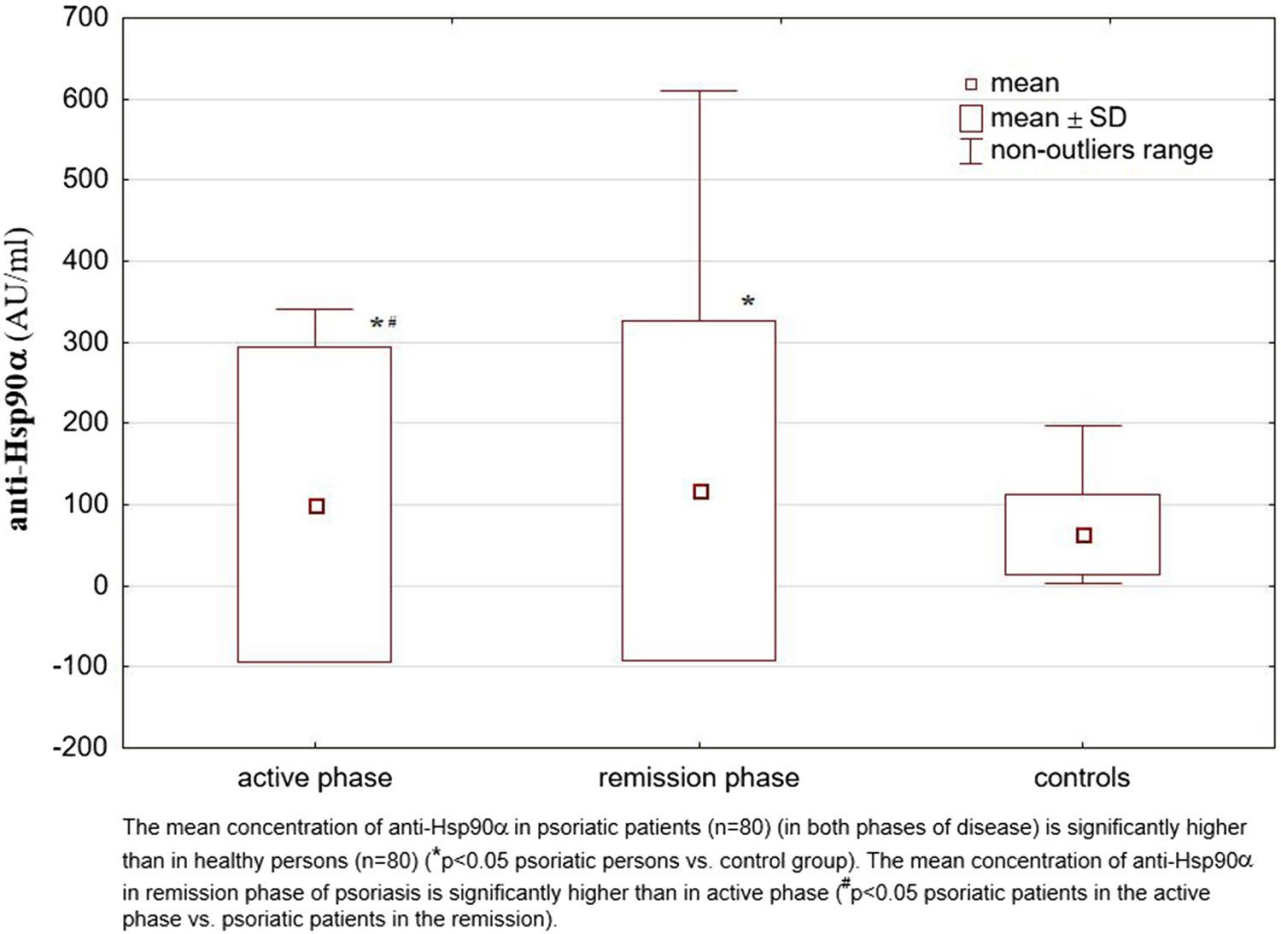
The concentrations of anti-Hsp90α antibodies in the sera of patients with psoriasis (in the active phase and in the remission phase of disease) and in healthy individuals (control group)

**Fig. 2:**
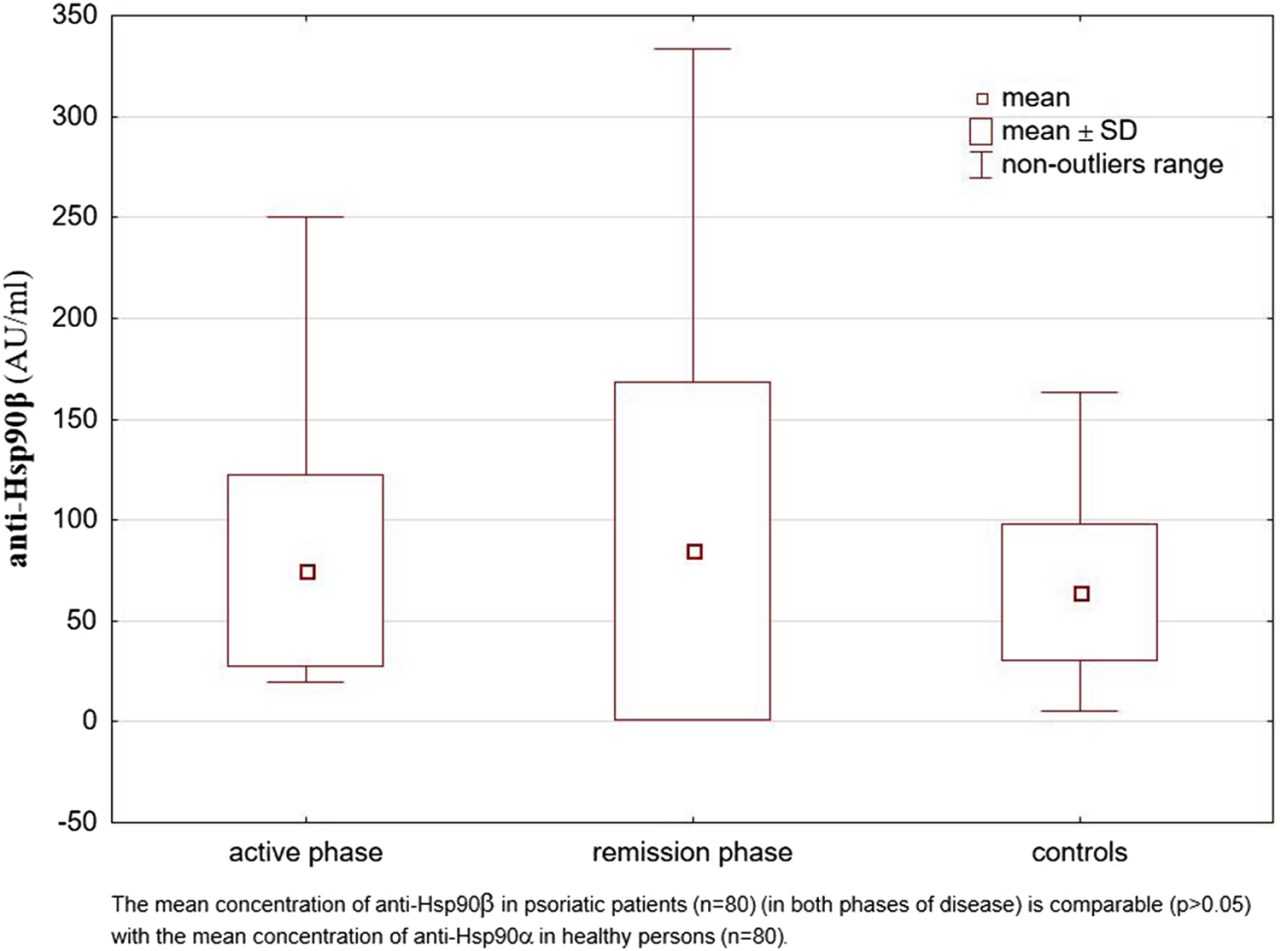
The concentrations of anti-Hsp90β antibodies in the sera of patients with psoriasis (in the active phase and in the remission phase of disease) and in healthy individuals (control group)

**Fig. 3:**
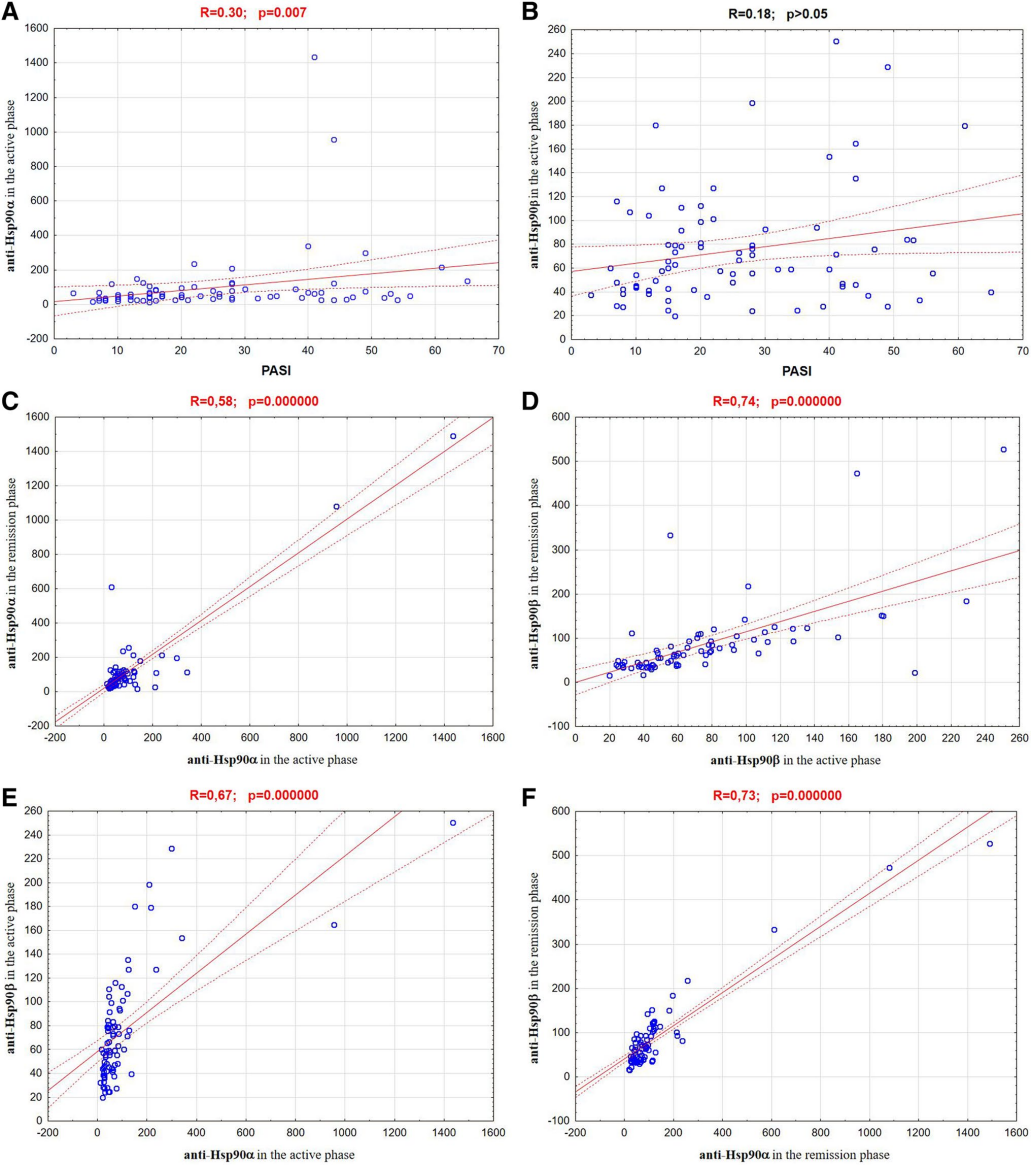

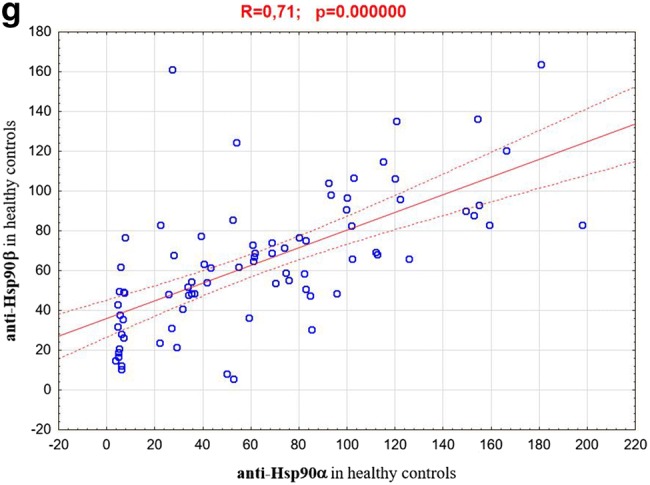
Correlation between: **a** anti-Hsp90α concentrations in the active phase of psoriasis and PASI values; **b** anti-Hsp90β concentrations in the active phase of psoriasis and PASI values; **c** anti-Hsp90α concentrations in the active phase of psoriasis and anti-Hsp90α concentrations in the remission phase; **d** anti-Hsp90β concentrations in the active phase of psoriasis and anti-Hsp90β concentrations in the remission phase; **e** anti-Hsp90α concentrations and anti-Hsp90β concentrations in the active phase of psoriasis; **f** anti-Hsp90α concentrations and anti-Hsp90β concentrations in the remission of psoriasis; **g** anti-Hsp90α concentrations and anti-Hsp90β concentrations in the healthy persons

Analysis of the obtained results showed that in the patients with psoriasis (both in the active phase of the disease and in the remission phase) concentrations of anti-Hsp90α antibodies were significantly higher than in healthy individuals. At the remission these concentrations were even higher than in the active psoriasis. However, mean concentrations of anti-Hsp90β antibodies in the psoriatic patients did not differ significantly from those observed for healthy individuals and these concentrations also did not differ between the active phase and remission phase of disease. The results are shown in Table 1 and illustrated in the Figs. 1 and 2. The concentrations of anti-Hsp90α antibodies in the active phase of disease correlated positively with values of PASI (*R* = 0.30; *p* = 0.007) (Fig. 3a). The analogous correlation for the concentrations of anti-Hsp90β antibodies and PASI was not demonstrated (*R* = 0.18; *p* > 0.05) (Fig. 3b). The concentrations of anti-Hsp90α antibodies in the active phase showed a moderate positive linear relationship with the concentrations of anti-Hsp90α antibodies in the remission phase of psoriasis (*R* = 0.58; *p* = 0.000000) (Fig. 3c) and the concentrations of anti-Hsp90β antibodies in the active phase showed a strong positive linear relationship with the concentrations of anti-Hsp90β antibodies in the remission phase of psoriasis (*R* = 0.74; *p* = 0.000000) (Fig. 3d). The concentrations of anti-Hsp90α antibodies correlated also positively with the concentrations of anti-Hsp90β antibodies, both in the active phase of psoriasis (*R* = 0.67; *p* = 0.000000) (Fig. 3e), and in the remission (*R* = 0.73; *p* = 0.000000) (Fig. 3f). The similar strong, positive correlation between concentrations of both tested antibodies was observed for healthy persons (*R* = 0.71; *p* = 0.000000) (Fig. 3g). In any of the groups the concentrations of anti-Hsp90α and anti-Hsp90β antibodies correlated with the age of studied individuals *(p* > 0.05).

**Table 1:** The concentrations of anti-Hsp90α and anti-Hsp90β antibodies in the sera of patients with psoriasis (in the active phase and in the remission phase of disease) and in healthy individuals (control group)

Examined parameters	Studied groups		
	Psoriatic patients in the active phase of disease (*n* = 80)	Psoriatic patients in the remission phase of disease (*n* = 80)	Controls (*n* = 80)
Anti-Hsp90α	100.23*^#^ ± 193.66	117.41* ± 208.81	63.56 ± 49.71
Anti-Hsp90β	74.96 ± 47.39	84.78 ± 83.78	64.13 ± 33.78

Values are (mean ± SD) (AU/mL)

*p < 0.05 psoriatic patients vs control group

^**#**^*p* < 0.05 psoriatic patients in the active phase of disease vs psoriatic patients in the remission

## Discussion

The induction of anti-Hsp immune response is dependent on their presence in the extracellular space. Intracellular localization, expression resulting from stress and specific functions within cells of most of Hsps are well known, but the origin and role of extracellular Hsps are less clear. Although they were originally suspected to be released from damaged, necrotic cells, their presence in healthy individuals may suggest that they have some regulatory functions in the circulation. The extracellular or cellular membrane-bound Hsps can also affect the inflammatory response which results in production of inflammatory cytokines and can participate in the immune response against other antigens (Kaźmierczuk and Kiliańska 2009; Wu and Tanguay 2006). The presence of anti-Hsps antibodies is observed under normal conditions, but it seems to increase under certain stress and pathological conditions. Such antibodies could regulate the Hsp-dependent processes, positively or negatively, and in this way they could be involved in pathogenesis of many diseases (Rowley and Karopoulos 1996; Wu and Tanguay 2006; Zugel and Kaufmann 1999).

One of the best known extracellular physiological function of Hsp90 is its role in innate and adaptive immunity—it takes part in antigen processing and presentation during immune responses (Basu and Srivastava 2000; Eustace and Jay 2004). Extracellular Hsp90 is also involved in wound healing (Li et al. 2012) and it is considered to be a key player in malignant invasion (angiogenesis, tumor cell motility and migration, cancer metastasis) (Sidera and Patsavoudi 2009; Tsutsumi and Neckers 2007). It seems that normal cells secrete Hsp90 into extracellular space in response to tissue injury or other factors disturbing the environment of their life, whereas tumor cells are characterized by secreting this chaperone for tissue invasion (Cheng and Li 2008; Li et al. 2012). The increased expression of anti-Hsp90 antibodies has frequently been found both in healthy individuals in different states related to the environmental stress exposure (e.g., under the influence of physical, chemical or biological environmental factors) and in various disease states (e.g., in autoimmune diseases, malaria, acute mania, graft-versus-host reaction, dermatitis herpetiformis, atherosclerosis or cancers) (Businaro et al. 2009; Goral et al. 2002; Hayem et al. 1999; Kasperkiewicz et al. 2014; Luo et al. 2002; Shen et al. 2006; Trieb et al. 2000; Wu and Tanguay 2006; Zhang et al. 2001).

The expression of extracellular form of Hsp90 in the skin, both healthy and pathologically changed, and immunization to Hsp90 during dermatological diseases are still not well known. Only few studies have been reported to date on the occurrence and possible role of Hsp90 and/or anti-Hsp90 antibodies in psoriasis. It was shown that under the influence of physical factors, e.g. hyperthermia (in vivo) (Wilson et al. 2000) and chemical factors, e.g., nickel salts (in vitro) (Carroll and Wood 2000) the expression of Hsp90 had increased, both in keratinocytes and fibroblasts. The PUVA therapy induced overexpression of Hsp90 only in vitro (not in vivo) (Al-Masaud et al. 1996). Kakeda et al. (2014) found that expression of Hsp90 was significantly increased in lesional psoriatic skin as compared with normal skin, nonlesional, and ustekinumab-treated psoriatic skin. Furthermore, using antibodies that recognizes both isoform of Hsp90, authors established that increased expression of Hsp90 in lesional psoriatic skin was the consequence of up-regulation only Hsp90α, but not Hsp90β. Interestingly, after successful treatment epidermal Hsp90α levels almost completely regressed to levels observed in nonlesional skin, whereas Hsp90β levels remained constitutively expressed. Moreover, significant increase of Hsp90^+^ cells (mainly mast cells) was showed in both the papillary and reticular dermis of lesional psoriatic skin compared with normal skin (Kakeda et al. 2014). Our results seem to confirm these observations. We showed that concentrations of anti-Hsp90α antibodies in sera of psoriatic patients were significantly higher in comparison to healthy controls sera, both in the active phase of disease and in the remission phase. In the remission of psoriasis concentrations of anti-Hsp90α antibodies were also considerably higher than in sera of the same patients in the active phase of disease, and concentrations in both disease phases correlated with each other significantly. However, the mean concentrations of anti-Hsp90β antibodies did not differ between psoriatic patients and control group.

Hsp90α is a protein highly inducible, its expression is induced by stress factors (inducible form/major form), whereas Hsp90β expression is thought to be constitutive (constitutive form/minor form). That is why Hsp90α expression is lower compared to Hsp90β expression in most cells (Sreedhar et al. 2004). The Hsp90β is the isoform of Hsp90 involved in normal cellular functions under physiological conditions, it is responsible for maintenance of nucleo- and cytoskeleton (Cambiazo et al. 1999; Sreedhar et al. 2003), cell differentiation (Liu et al. 1999) and cytoprotection (Sreedhar and Csermely 2004). The expression of this isoform can also be induced, but only under long-term stress conditions and during long-term cellular adaptation (Sreedhar et al. 2004), which is associated with the development of drug resistance (Bertram et al. 1996) and slow progression of chronic tumors (Ogata et al. 2000). In contrast to Hsp90β, expression of Hsp90α can even be induced by short-term stress (Sreedhar et al. 2004), and its high expression was shown to be associated with various tumor progression (Gress et al. 1994; Yufu et al. 1992), enhanced cell cycle regulation (Jerome et al. 1993), and growth factor-mediated signal transduction via tyrosine kinases (Jerome et al. 1991). This isoform is also involved in induction of apoptosis (Wu et al. 2002). In summary, increased level of Hsp90α correlates with fast response to stress, while increased level of Hsp90β—with long-term cellular adaptation to non-physiological conditions (Sreedhar et al. 2004). Therefore, the obtained results can suggest that in psoriatic patients extracellular expression of Hsp90α is increased and as a consequence the immunization against this isoform is increased as well. Elevated levels of the Hsp90 which correlate with levels of autoantibodies to Hsp90 have already been observed in patients with systemic lupus erythematosus (Ripley et al. 2001).

Still it remains unclear whether high extracellular expression of Hsp90α and/or anti-Hsp90α immunization is a cause or a result of the processes taking place in involved psoriatic skin. Do the Hsp90α and/or anti-Hsp90α antibodies take part in skin lesions arising or are there the non-physiological conditions in psoriatic skin that cause increased Hsp90α expression and/or anti-Hsp90α immunization? The answers to these questions are very difficult, so far. The presence of positive correlation between the anti-Hsp90α concentrations and PASI values can point to the second possibility. On the other hand, significant increase of anti-Hsp90α antibodies which accompanies remission of the disease can suggest either increase of immunization in time (if the increase of Hsp90α expression is a sequence of psoriatic lesions) or increase of immunization as a result of treatment (if the increase of Hsp90α expression during treatment can be considered as a defensive reaction of pathologically changed skin cells against the drugs impact). The third possibility is favorable role of anti-Hsp90α antibodies in the process of psoriatic plaques recovery (if the increase of Hsp90α expression plays an etiopathological role in psoriasis development).

The studies indicating the role of Hsps in activation and maturation of skin dendritic cells and in induction of psoriatic lesions have been published in recent years. Boyman et al. (2005) observed markedly increased presence of dendritic cells expressing common Hsp receptor CD91 juxtaposed to lesional keratinocytes expressing Hsp70 during induction of psoriasis. In marked contrast, CD91^+^ dendritic cells were present at low numbers in normal skin and symptomless psoriatic skin. In vitro CD91^+^ dendritic cells activated by Hsp70 expressed tumor necrosis factor (TNF)-*α*—an important proinflammatory cytokine in the immunopathogenesis of psoriasis (Boyman et al. 2005). The keratinocytes can be significant source of Hsp70 in psoriasis, more important than fibroblasts, macrophages or lymphocytes (Dong et al. 2011). Curry et al. (2003) showed high concentration of heat shock proteins: Hsp27, Hsp60, and Hsp70 in psoriatic lesions and high expression of CD91^+^ dendritic cells in the upper dermis directly under dermal–epidermal junction. It was also observed (in vitro) that Hsps-induced dendritic cells maturation and stimulated them to interleukin (IL)-12 production, what could contribute to the Th1 cell-mediated reaction causing transition of symptomless to lesional psoriatic skin (Curry et al. 2003). The results of the quoted studies suggest the role of Hsps and dendritic cells in lymphocytes differentiation and inflammatory infiltration and point to the significance of the dynamic interplay between innate and adaptive immunity in chronic inflammation accompanying diseases such as psoriasis (Stebbing et al. 2005).

Based on the previous results (Kakeda et al. 2014) and our observations one could formulate a hypothesis that the similar role of activator and inductor of psoriatic lesions plays secreting Hsp90α by keratinocytes. Keratinocytes are thought to play a major role both in initiating psoriatic inflammation in the context of skin trauma and in amplifying psoriatic inflammation during maintenance of lesions (Lowers et al. 2013). Hsp90α released by stressed keratinocytes activates [by CD91 receptor—a common receptor for all Hsps, also Hsp90 (Basu et al. 2001; Stebbing et al. 2003)] dendritic cells leading to their migration, antigen presentation, and secretion of proinflammatory cytokines (Boyman et al. 2005). One of these cytokines—IL-23 stimulates Th17 cells to releasing IL-17 and IL-22, which induce keratinocyte hyperproliferation. Another cytokine TNF-*α* induces secretion of Hsp90α from keratinocytes. This self-amplifying mechanism represents the chronic stage of psoriatic inflammation and Hsp90α likely plays a crucial role in this loop perpetuation (Kakeda et al. 2014). Also mast cells in dermis express and release Hsp90α which may lead to further activation of CD91-expressing antigen-presenting cells in psoriatic lesions (Kakeda et al. 2014). Thus, the anti-Hsp90α immune response and anti-Hsp90α autoantibodies, by blocking its activity, could favor the recovery of psoriatic lesions (in the remission phase the concentrations of anti-Hsp90 antibodies are higher than in the active phase of disease). On the other hand, the mean concentrations of Hsp90β, whose expression is thought to be constitutive, do not differ between psoriatic patients and healthy individuals and that is why concentrations of anti-Hsp90β antibodies are also comparable and in psoriatic patients do not correlate with PASI values.

The increased concentrations of anti-Hsp90α antibodies were observed, among others, in patients with heat stroke (the concentrations of anti-Hsp90β antibodies did not differ from concentrations in control group) (Wu et al. 2001). It was confirmed that only isoform *α* of Hsp90 (and not β) played extracellular role in cancer cell invasiveness and tumor metastasis by matrix metalloproteinase-2 activation and tumor angiogenesis induction (Eustace et al. 2004; Sims et al. 2011; Song et al. 2012). It was also revealed that the secretion of Hsp90α (but not Hsp90β) was increased in activated endothelial cells which promoted their angiogenic activities, whereas Hsp90α neutralizing antibodies reversed this effect and the extracellular Hsp90α induced angiogenesis during wound healing (Song and Luo 2010). In wound healing process the extracellular Hsp90α also promotes dermal fibroblasts migration (Li et al. 2007). The angiogenesis and migration of inflammatory cells from blood vessels are also the first histopathological symptoms of arising psoriatic plaque. Perhaps, the extracellular Hsp90α also plays a role of psoriatic lesions initiator through its effect on vascular activity and cell motility. The immune response against Hsp90β in psoriasis does not seem to be different from one observed in healthy controls. However, it is worth to take note of the presence of very high anti-Hsp90β antibodies concentrations in single patients. Anti-Hsp90β concentrations (similarly to anti-Hsp90α concentrations) correlate with each other in active phase and in remission phase, although (differently than in case of anti-Hsp90α concentrations) obtaining the remission does not cause the significant increase in the concentration of these antibodies. The explanation of these results remains difficult, but it does not permit to exclude the possibility of immunization by this isoform of Hsp90 in psoriasis. It was suggested that only the immune response against Hsp90β could be of importance in pathogenesis of such diseases as multiple sclerosis (Cid et al. 2007a, b) or autoimmunological ovarian infertility (Pires and Khole 2009).

The observed correlations of anti-Hsp90α and anti-Hsp90β concentrations seem also interesting. These concentrations correlate positively in healthy persons and their values are comparable. It can suggest the similar level of immunological response to both Hsp90 isoforms in physiological conditions. In psoriatic patients, the concentrations of anti-Hsp90α and anti-Hsp90β antibodies also correlate significantly, both in the active and in the remission phase. It may indicate that the immunization against β isoform also is present during formation and remission of psoriatic lesions, nevertheless it not so strong against α isoform.

This is the first report showing the presence of anti-Hsp90α and anti-Hsp90β antibodies in psoriatic patients in the active phase and in the remission phase of the disease. There is a need of further studies to verify the Hsp90α extracellular role in psoriatic lesions as well as to identify the “stressor”, which initiates this chaperone expression. The confirmation of the extracellular Hsp90α role in etiopathogenesis could create a new therapeutic and preventive possibilities in psoriasis. The anti-Hsp90 therapies have been tested in various inflammatory and autoimmune disease models and in many of them they have shown their effectiveness (reviewed by Tukaj and Węgrzyn 2016). Also Debio 0932—a new oral Hsp90 inhibitor developed for anti-cancer therapy alleviated psoriasis in a xenograft transplantation model (Stenderup et al. 2014). Perhaps the blockers of CD91 receptor, Hsp90α inhibitors or anti-Hsp90α antibodies will be effective anti-psoriatic drugs in the future.

In conclusion, the obtained results indicate an existence of increased immunological response to Hsp90α in psoriasis. It may suggest the role of the extracellular form of this chaperone and/or anti-Hsp90α antibodies in etiopathogenesis of this dermatosis. Inhibition of Hsp90α may represent a more specific strategy than the general anti-Hsp90 therapeutic approaches.
